# Neuroimaging, Biomarkers, and Management of Dementia with Lewy Bodies

**DOI:** 10.3389/fneur.2013.00151

**Published:** 2013-10-07

**Authors:** Hasmet A. Hanagasi, Başar Bilgiç, Murat Emre

**Affiliations:** ^1^Behavioral Neurology and Movement Disorders Unit, Department of Neurology, Istanbul Faculty of Medicine, Istanbul University, Istanbul, Turkey

**Keywords:** Lewy body disease, dementia, Lewy bodies, magnetic resonance imaging, biomarkers

## Introduction

Dementia with Lewy Bodies (DLB) is a neurodegenerative disorder that affects cognition, behavior, movement, and autonomic function. Along with a gradually developing dementia, it is characterized by fluctuating cognition, spontaneous parkinsonism, and recurrent visual hallucinations. This article provides a brief overview of neuroimaging, biomarkers, and management in DLB.

## Neuroimaging and Other Auxiliary Investigations

In structural imaging with MRI, relative preservation of hippocampus and medial temporal lobes as compared to atrophy in these regions in patients with AD patients have been described in pathologically confirmed DLB patients (1). Sabattoli et al. found that there was mild hippocampal atrophy (10–20%) in DLB patients compared to control group, but it was less than seen in AD (2). There is, however, considerable overlap between AD and DLB, and the utility of MRI for differential diagnosis is limited. In a recent study, Lewy Body (LB), but not AD-type pathology was associated with reduced amygdala volume in pathologically verified DLB cases, but neither LB nor AD pathology was associated with volume loss in hippocampus or entorhinal cortex, suggesting other neuropathological factors accounting for atrophy in these structures (3). Others suggested that hippocampal atrophy on MRI may be associated with neurofibrillary tangles in patients with LB pathology (4). Atrophy in other cortical and subcortical structures has also been reported in DLB, including striatum, substantia innominata, hypothalamus, and dorsal midbrain (4). The rate of overall cerebral atrophy in longitudinal MRI studies has been reported to be 1.4% per year, three times more than that seen in controls, but less than that seen in AD (2% per year) (5).

In a Diffusion Tensor Imaging study, changes [increased diffusion (*D*) and decreased fractional anisotropy (FA) values] in corpus callosum and pericallosal areas were found in DLB patients as compared to normal controls (6), white matter was also affected in the frontal, parietal, and occipital areas. In another study DLB group had reduced FA in the precuneus area compared to controls and AD patients (7). Areas of reduced FA in DLB vs. controls were also found primarily in parietooccipital white matter tracts whereas the changes were more diffuse in AD; compared to AD, DLB was also associated with reduced FA in pons and left thalamus (8). This study suggests that pattern of DTI changes in AD and DLB are different. Resting state fMRI studies suggested increased functional connectivity between the right posterior cingulate and other brain areas (9).

SPECT and PET studies have demonstrated decreased glucose metabolism and perfusion deficits in parietal and occipital areas in DLB (10, 11). However, occipital hypometabolism is not always present and FDG-PET changes may be similar to that seen in AD. Reduced uptake of 18 F-fluorodopa in the striatum may distinguish DLB from AD with high sensitivity and specificity (12). Several studies, using 123-I-beta-CIT SPECT, demonstrated reduced dopamine transporter binding in the caudate and posterior putamen in DLB compared to AD patients (13, 14), but no difference between DLB and Parkinson’s Disease (PD) patients. Similarly, thoracic SPECT imaging using 123-I metaiodobenzylguanidine (MIBG), a marker of post-ganglionic cardiac sympathetic innervation, shows reduced cardiac MIBG uptake in DLB and PD patients as opposed to normal findings in AD (15). It was suggested that combining SPECT and MIBG scintigraphy could increase the diagnostic accuracy for DLB (16). Pittsburgh compound B (PiB) is an *in vivo* indicator of β-amyloid load, 80% of DLB cases were found to have increased amyloid load in 11C-PIB PET, whereas only 20% of patients with Parkinson’s Disease Dementia (PD-D) have this finding (17).

Standard EEG may show early slowing of background activity in DLB patients compared with AD as well as epoch by epoch fluctuations. Frontal intermittent delta activity and transient temporal slow waves are other changes which are more common in DLB than in AD (18). Video-polysomnography, including surface electromyography (EMG) of the chin and extremities is necessary for the diagnosis of RBD, which is a common feature of DLB.

## Cerebrospinal Fluid Biomarkers in DLB

Currently, there are no blood or CSF markers which can be used for diagnosis, to follow disease progression or as an outcome parameter for therapeutic interventions in DLB. Alpha-synuclein has been studied as a potential biomarker for DLB, but results have been controversial (19, 20). Amyloid beta 40–42 in CSF is a biomarker for AD, several studies reported lower amyloid beta 40–42 levels also in DLB compared to controls and PD-D cases (21, 22). In addition, Mulugeta et al. suggested CSF amyloid-38 as a diagnostic biomarker for DLB (23); A-42/A-38 ratio discriminated AD from DLB with a sensitivity of 78% and a specificity of 67%.

## Management of Patients with DLB

Management of DLB includes both non-pharmacological and pharmacological approaches. Non-pharmacologic approaches have the potential to reduce clinical symptoms and functional impairment. Recognition of potentially treatable sensory impairments such as impaired vision and environmental changes such as improving lighting may reduce hallucinations and falls. Behavioral symptoms may be relieved or reduced by education and behavioral modifications of caregivers.

All drugs with anticholinergic effects, e.g., tricyclic antidepressants, anticholinergics, antispasmodics should be discontinued as they have the potential to further impair cognition, exacerbate psychotic symptoms, and may be associated with orthostatic hypotension in patients with DLB (24). l-DOPA should be preferred to treat motor symptoms, it should be started at low doses and increased slowly to the minimum required dose. Patients with DLB may be less responsive to levodopa therapy than those with Parkinson’s disease. Only 30–50% of patients improve significantly (25, 26). Other antiparkinsonian agents, including monoamine oxidase B inhibitors, amantadine, and dopamine agonists are usually less tolerable in DLB patients.

Orthostatic hypotension can be treated with hydration, increased dietary salt intake or with salt tablets, avoidance of prolonged bed rest, thigh-high compression stockings, efforts to stand up slowly, and avoidance of medications that contribute to orthostasis. If these measures are ineffective, fludrocortisone or midodrine can be considered. REM sleep behavior disorder can be treated with low dose clonazepam (0.25–1.0 mg) at bedtime, however clonazepam has the potential to worsen the symptoms of obstructive sleep apnea (27). Melatonin may also be effective at a dose of 3–12 mg, either as monotherapy or in conjunction with clonazepam. Although evidence base in DLB is weak, drugs such as modafinil and methylphenidate can be considered to treat excessive daytime sleepiness.

Visual hallucinations are often the most troubling neuropsychiatric feature in DLB, they do not require drug treatment if they are not frightening. Atypical antipsychotics are used to treat psychotic symptoms, but they should be used with great caution in DLB, as some patients may show severe neuroleptic sensitivity which is not predictable in an individual patient (28). In addition, antipsychotics may increase the risk of cerebrovascular events and death in elderly patients with dementia (29). As reductions in cholinergic activity correlate with hallucinations, cholinesterase inhibitors may be considered for management of hallucinations and delusions, if they are not severe, before using a neuroleptic (30, 31). If the symptoms are refractory, cause significant impairment or severe burden for caregivers, quetiapine, and clozapine can be considered. Initial doses should be low and the dose should be titrated slowly while monitoring for functional decline. Neuroleptics can cause orthostatic hypotension and blood pressure monitoring may be necessary in some patients.

Patients with DLB have severe cholinergic deficits. Results from randomized controlled trials with cholinesterase inhibitors rivastigmine and donepezil indicated improvements in cognitive function and behavioral symptoms (30, 31). McKeith et al. reported a multicenter, randomized, controlled study of 120 patients with DLB using rivastigmine 6–12 mg/day or placebo for 20 weeks (30). Although there was a slight improvement in mean MMSE score in the rivastigmine group at week 20, this was not statistically significant compared to placebo nor was the clinician-assessed global status. Change from baseline in mean Neuropsychiatric Inventory (NPI, 10 items) score was not significantly different between the two groups in the intention-to-treat, but statistically significant in favor of rivastigmine in the observed cases analysis. More than twice as many patients on rivastigmine (63.4%) than on placebo (30.0%) showed at least a 30% improvement from baseline in their NPI-4 scores (*P* ≤ 0.001). Apathy, anxiety, delusions, and hallucinations were the symptoms to show most response. There was also a significant improvement in a computerized test of attention, choice reaction time. Improvements seen with rivastigmine returned to baseline during a 3-week washout period. Nausea (37%), vomiting (25%), anorexia (19%), and somnolence (9%) were the most common side effects. Worsening of parkinsonism was not reported, although emergent tremor was noted in four rivastigmine-treated patients.

In another large randomized, placebo controlled study the effect of donepezil (3, 5, 10 mg/day) was assessed in 140 DLB patients for 12 weeks (31). Patients given 5 or 10 mg donepezil showed greater improvement in the majority of the cognitive and behavioral scales, including MMSE and NPI. Donepezil treatment also led to improved global function, as measured by CIBIC-plus and reduced caregiver burden at the highest dose. Patients taking donepezil were less apathetic, less anxious, had less cognitive fluctuation, fewer delusions and hallucinations compared to placebo patients. Approximately 8% of the study population withdrew due to adverse effects, and the prevalence of withdrawal or adverse effects, including typical cholinergic side effects, did not differ among treatment groups.

Changes in glutamatergic activity have been reported in patients with DLB (32). Uncompetitive *N*-methyl-d-aspartate antagonist memantine was tested in two randomized placebo controlled studies including DLB patients; the results were however not consistent (33, 34). The larger study in patients with Lewy-body-related dementias included 199 patients, of which 121 had PD-D and 78 had DLB (34). At week 24, patients treated with memantine had greater improvements in global status [as measured by clinical global impression of change (CGIC) score] than did patients who received placebo; improvements were predominantly in the DLB group. Behavioral symptoms as assessed with NPI total score significantly improved in the DLB group only. Cognitive test scores, activities of daily living scores, motor symptoms, or caregiver burden scores did not show significant improvements in either patient group. Adverse events in the two treatment groups were similar, except for slightly more sedation in the memantine treated patients. In another, smaller randomized trial of memantine in patients with Lewy-body-related dementias, including both patients with DLB or PD-D significantly improved mean CGIC score was observed in the total population after 6 months of treatment, the difference was driven by a larger efficacy in the PD-D group (33). NPI scores showed a statistically significantly improvement in the memantine group as compared to placebo in the DLB group, but not in the PD-D population. No statistically significant differences were observed in individual cognitive tests, ADCS-activities of Daily Living or Zarit caregiver burden scores. The incidence of adverse events and number of discontinuations due to adverse events were similar in both groups.

In summary, cholinesterase inhibitors such as rivastigmine and donepezil should be considered in all patients with a diagnosis with DLB, taking into account potential benefits and risks. The data on benefits of memantine are less clear, memantine may be considered in appropriate patients with prominent behavioral symptoms.

## Conclusion

Structural and functional imaging may help in the diagnosis and differential diagnosis of DLB, there are however no unique patterns, SPECT imaging of dopamine transporter can differentiate from AD. Currently there is no validated biomarker for DLB, an important target for future research is to improve the accuracy of diagnosis by developing sensitive biomarkers. Cholinesterase inhibitors remain the first choice therapy for cognitive impairment. There are as yet no disease-modifying agents, new treatments are urgently required for DLB which is second most frequent degenerative dementia following AD.
